# Distributions and trends in sexual behaviors and HIV incidence among men who have sex with men in China

**DOI:** 10.1186/1471-2458-12-546

**Published:** 2012-07-24

**Authors:** Lei Zhang, Eric P F Chow, David P Wilson

**Affiliations:** 1 The Kirby Institute, Faculty of Medicine, University of New South Wales, Sydney, NSW 2052, Australia

**Keywords:** Men who have sex with men, MSM, China, HIV, Incidence, Sexual behaviors

## Abstract

**Background:**

HIV prevalence is increasing at a concerning rate among men who have sex with men (MSM) in China. Numerous studies have reported on levels of behaviors of Chinese MSM for different types of sexual partnerships, such as regular, non-commercial casual and commercial. This study aims to investigate the trends HIV incidence rates in relation to their risk sexual behaviors and partnership types among Chinese MSM.

**Method:**

Through a comprehensive literature research from available English and Chinese literature databases, we collated relevant information of sexual behaviors of Chinese MSM. Further, with the utilization of a mathematical optimization approach, this study reconciles the distributions of sexual behavioral data over the last decade and infers the heterogeneous distributions of behavioral patterns among Chinese MSM. Distributions of high-risk behavioural indicators, including the number of sexual partners, number of sexual acts and condom usage in the past 6 months, are calibrated to available empirical data. Based on the resultant temporal trends in these distributions, the trends in HIV incidence rates associated with each type of partnership among MSM in China are also estimated.

**Results:**

A total of 55 qualified articles have been identified. An average MSM has approximately 0.96 (95% CI, 0.59-1.18) regular, 3.75 (1.72-6.25) casual and 1.61 (0.97-2.78) commercial partners over a 6 month period and 4.33 (2.81-6.46), 1.42 (0.62-3.08), 1.48 (0.79-3.30) sexual acts per partnership respectively, corresponding to a total of 11.87 (8.87-15.25) acts. Condom usage has increased significantly during 2002–2010, at annual increases of 3.58% (2.98-4.12%), 5.55% (4.55-6.54%), and 5.03% (4.19-5.74%) for regular, casual and commercial partners respectively. These behavioral data implies an increase in HIV incidence of approximately 3.3-fold, from 2.04 (1.96-2.12) to 7.02 (6.71-7.35) per 1000 person-years during the same period. The proportion of new infections attributed to regular partnerships increased from 34% to 40%, whereas infections attributed to commercial partnerships reduced from 29% to 23% during 2002–2010.

**Conclusion:**

Regular partnerships are the main contributor of new HIV cases among MSM in China, public health intervention strategies are required to increase condom usage and HIV testing rates among regular partners to curb the growing trend HIV incidence.

## Background

By 2009, an estimated 0.74 (0.56–0.92 million) people in China were living with HIV [1]. In contrast to other regions around the world, China has a relatively low HIV prevalence of approximately 0.06% among the general adult population [1,2]. In recent years sexual transmission, especially homosexual transmission between men, has become the primary mode of transmission [1,3]. National statistics has revealed that the proportion of new HIV annual infections due to male-to-male sexual intercourse increased from 12% in 2007 to 33% in 2009 [1]. The estimated national HIV prevalence among men who have sex with men (MSM) has tripled, from 1.4% to 5.3%, during 2001–2009 [4]. Accumulating evidence indicates that MSM in China have a much higher risk of acquiring HIV than other population groups [5]. The rapid increase in HIV prevalence among Chinese MSM is likely due to their high-risk sexual behaviors and multiple partnerships.

Currently, there are approximately 5–10 million MSM in China [6], and approximately 95% of whom had at least one male sexual partner in the past six months [7]. Sexual partners among MSM can be categorized into three main types: (1) regular; (2) non-commercial casual and (3) commercial. It is well-known that multiple sexual partners can increase the risk of HIV acquisition and high-risk sexual behaviors such as unprotected penile-anal sexual intercourse can be a major risk factor for HIV transmission [8]. In the United States, unprotected sexual intercourse among regular MSM partnerships contribute to the majority of new HIV infections [9]. In comparison, little is known about how sexual intercourse among different sexual partnerships among MSM has contributed to new HIV cases in China. Although reported levels of consistent condom use among MSM have been increasing in China [10], the relative level and change in condom usage among different partner types also remains unclear. Therefore, this study aims to estimate HIV incidence among MSM by type of sexual partner, by considering the distribution of sexual partner rates and condom usage.

Through a comprehensive research of published literature, we collate all available sexual behavioral data from these studies to describe the overall behavioral patterns of Chinese MSM and temporal trends. We then employ a mathematical optimization routine to ‘reconcile’ the distributions of sexual behavioral data over the last decade in order to infer the heterogeneous distributions of behavioral patterns that exist among the population of Chinese MSM. Based on these distributions and a transmission risk equation, HIV incidence rates associated with each type of partnership are estimated. We then discuss the risk of HIV transmission among each sexual partnership type and its implications to HIV control and prevention among Chinese MSM.

## Method

### Search strategy and data collection

A comprehensive literature search for published peer-reviewed studies was performed independently by two investigators (EPFC, LZ) from the following electronic databases: PubMed, Wanfang Data, China National Knowledge Infrastructure (CNKI) and Chinese Scientific Journals Fulltext Database (CQVIP), from 2001 to May 2011. The keywords searched in the databases included (“human immunodeficiency virus” OR “HIV” OR “Acquired immune deficiency syndrome” OR “AIDS”) OR (“homosexual” OR “MSM” OR “men who have sex with men” OR “gay”) OR (“behaviors” OR “attitudes” OR “high-risk behaviors” OR “condom” OR “partners”) AND (“China”). The references in the relevant articles were searched manually. We limited our search to articles published in Chinese and English only.

Studies were included if they: (1) reported the percentage of consistent condom use in the past six months with any types of male partners; or (2) reported the number of different types of sexual partners in the past six months; or (3) reported the number of sexual acts in the past six months with different types of male partners; through a peer-reviewed publication of cohort or cross-sectional study. Review papers, non-peer reviewed theses, local reports, conference abstracts and presentations were excluded from this study. Studies which did not report study location, time periods and sample size were also excluded. For duplicate studies with the same source, studies published in Chinese or published latest were excluded. The following information were extracted from eligible studies: first author and year of publication, study period, study location, average and range of ages of MSM in the study, recruitment method, sampling method, sample size, and types of male sexual partners (Additional file 1: Table S1-3). However, we only included studies with at least 2 data points on the distribution of number of partners or acts.

### Fittings and optimization of MSM sexual behaviors

**Figure 1:**
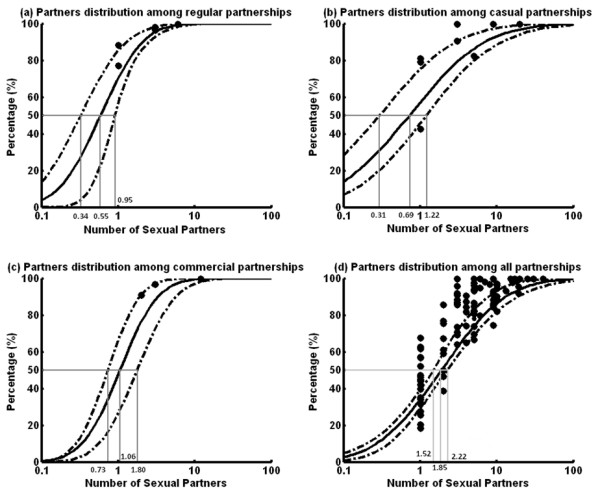
The distribution of the number of (a) regular; (b) casual; (c) commercial and (d) all sexual partners among Chinese MSM in the past 6 months during 2002–2010.

**Figure 2:**
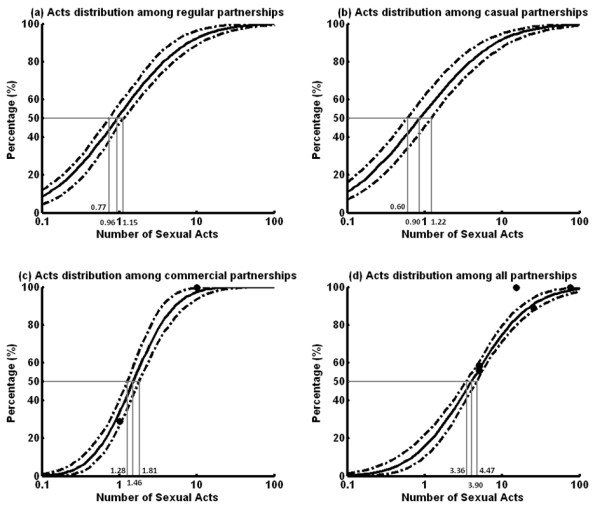
**Distribution of number of acts among (a) regular; (b) casual; (c) commercial; and (d) all partners among Chinese MSM in the past six months during 2002–2010. **Notably, since we assume that the distribution of the overall number of sexual acts is the sum of the corresponding lognormal distributions from the separate types of sexual partnerships, this enables us to estimate the distribution of the number of acts in regular sexual partners and casual partners even in the absence of empirical data. Large uncertainties for absent distribution are included for these indicators.

The optimization methodology has been previously published [11]. Briefly, lognormal distributions represent probability distributions of a random variable whose logarithmic values are normally distributed; these statistical distributions have been accepted as a good approximation to the distribution of sexual partners and acts of human populations [12]. Due to expected substantial heterogeneity across studies caused by different geographical location and time with various study methods, we employed an optimization approach to reconcile the available sexual behavioral data, that is, the distribution of the number of sexual partners and acts. Four types of sexual partnerships (regular, non-commercial casual, commercial and overall) in the past 6 months are reported in the published literature. We make the following assumptions: (1) if a study does not specify any sexual type, the reported distribution is assumed to represent the overall distribution; (2) the distribution of the overall number of sexual partners (or acts) is the sum of the corresponding lognormal distributions from the separate types of sexual partners, which are also approximated by lognormal distributions. Notably, this approximation of addictive distributions enables us to estimate the distribution of the number of sexual partners (or acts) in any type of partnerships even in scenarios where empirical data is completely absent (Figure 1 and 2), as long as the overall partners (or acts) distributions are available. The lognormal distribution assumption is also important for the subsequent optimization exercise, where it fits empirical data to the distribution without arbitrarily imputing missing data; (3) non-commercial casual partnerships are commonly ‘one-off’, suggesting similar distributions of numbers of casual sexual partners and acts. In such cases, large uncertainties for absent distribution are included in the optimization procedure. The optimization routine minimizes the difference between the optimized fits and the actual datasets with the error function:

$$E=\Sigma_{i}{\left(N_{i}-D_{i}\right)}^{2}\text{,}$$

where, $N_{i}∼LN(\mu_{i},\delta_{1})$ denotes curves of lognormal distributions of sexual partners with each sexual type *i*, mean *μ*_*i*_ and variance *δ*_*i*_, and *D*_*i*_ denotes a randomly generated bootstrapped dataset of the original datasets for the corresponding sexual type. Thus the error function represents the sum of differences between the optimized distribution curves and bootstrapped data points. Bootstrapping is performed by re-sampling on the original datasets to generate a new set of datasets with identical dimensions as the original datasets for each simulation. The weighted re-sampling process is designed in a way that the probability a dataset being re-sampled is proportional to its sample size. This ensures studies with larger sample size weights proportionally during optimization. The optimization routine is repeated 100 times and the best 50 fits are selected to provide uncertainty bounds. This optimization route was performed on a linear regression model of the temporal trend of condom usage with estimated 95% confidence intervals. Uncertainty bounds are reported as 95% confidence intervals in parentheses. The mean number of sexual partners and acts, together with their 95% confidence intervals, are hence calculated numerically based on the fitted lognormal distribution with the analytical expression $e^{\mu +\sigma 2/2}$.

The distribution of six-monthly incidence rates of HIV infection for each sexual partnership type is calculated based on distributions of number of sexual partners ($N_{i}∼LN(\mu_{i},\delta_{i})$), number of sexual acts ($S_{i}∼LN(\mu_{i},\delta_{i})$), and condom usage ($C_{i}∼N(\mu_{i},\delta_{i})$) in the past 6 months, according to the following mathematical expression (which is a relatively standard force of infection term in transmission modelling, using a Bernoulli equation):

$$\overset{\begin{array}{c} \text{HIV incidence rate }\\ \text{in sexual partnership }\\ \text{with type }i \end{array}}{\overset{︷}{I_{i}(t)}}=p(t)S_{i}(1-\overset{\begin{array}{l} \text{Transmission probability }\\ \text{of HIV infection per }\\ \text{protected penile-anal act} \end{array}}{\overset{︷}{{\left(1-(1-\varepsilon )\beta \right)}^{N_{i}(t)C_{i}(t)/S_{i}(t)}}}\overset{\begin{array}{l} \text{Transmission probability }\\ \text{of HIV infection per }\\ \text{un-protected penile-anal act} \end{array}}{\overset{︷}{{\left(1-\beta \right)}^{{}^{N_{i}(t)(1-C_{i}(t))/S_{i}(t)}}}})$$

The estimated annual HIV incidence among MSM is hence the sum of incidence contributed by each sexual type. Here, *p*(*t*) denotes the HIV prevalence among Chinese MSM during 2001–2010, the prevalence data is extracted from a recent publication that synthesized the available HIV prevalence data in China during 2001–2010 in a meta-analysis [13]. This study reported that HIV prevalence among Chinese MSM increased from 1.3% (95% CI: 0.8–2.1%) prior to 2004 to 2.4% (95% CI: 1.7–3.2%) during 2005–2006 and then to 4.7% (95% CI: 3.9–5.6%) after 2007. The per-act infectivity *β,* for homosexual penile-anal sex was assumed to be 1–1.5% [8], whereas the condom efficacy ε, was assumed to be 85-95% [14-18]. Based on the optimization routine, each simulation will generate a unique set of distribution of number of sexual partners and acts and condom usage. The calculation of the above Bernoulli equation takes into account of these distributions and other biological parameters. This results in a distribution of HIV incidence. The calculation is repeated for 50 times to estimate the confidence intervals of incidence distribution. Annual HIV incidence was calculated by multiplying the six-monthly incidence estimates by two. We define incidence rates above 20 cases per 1000 persons as high incidence rates and use this threshold to assess the temporal trend of HIV epidemic trend among Chinese MSM.

## Results

### Literature search and selection

We identified 635 articles from one English and three Chinese electronic databases (103 in PubMed, 175 in CQVIP, 131 in CNKI and 226 in Wanfang); we also identified 8 extra articles from the reference lists of the selected articles. After screening the titles of articles, we excluded 335 articles due to duplication of articles from multiple databases. We screened the abstracts of the remaining 308 articles, following which 43 articles were excluded because 5 were unrelated and 38 were non peer-reviewed. The remaining 265 articles were eligible for full-text screening; we further excluded 210 articles (5 were review papers, 10 did not report the study site, 37 did not report the types of partners in condom usage, 64 did not report the MSM behavioral data in the past six months and 94 did not report any behavioral data). Finally, 55 articles (13 in English and 42 in Chinese) were included in qualitative analysis and subsequent optimizations (33 studies reported the numbers of partners, 24 studies reported levels of consistent condom use among different types of partnerships and 4 studies reported the number of sexual acts with different types of partnerships, Additional file 1: Figure S1).

### Sexual behaviors among Chinese MSM

**Figure 3:**
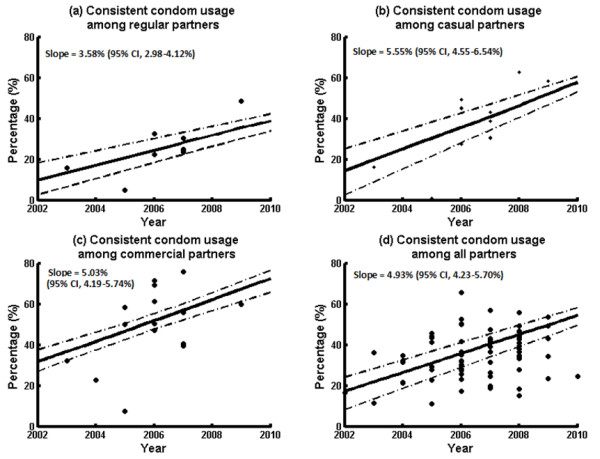
Consistent condom usage among (a) regular; (b) casual; (c) commercial partners in the past six months among Chinese MSM during 2002–2010.

A lognormal distribution function was used to obtain mean and median values of partner distributions of individual studies (Additional file 1: Table S1). Spearman correlation tests were performed on the mean and median of number of sexual partners (and acts) with their studied years whenever data were sufficient, but none of the correlations were found to be statistically significant (Additional file 1: Table S1-3). In contrast, condom usage levels among MSM were shown to be significantly increasing over time among all sexual types . Condom usage increased significantly at annual rates of 3.58% (2.98-4.12%), 5.55 (4.55-6.54), 5.03% (4.19-5.74%) for regular, casual and commercial partners respectively during 2002–2010 (Figure 3a-c).

Since distributions in numbers of sexual partners and acts did not vary temporally, this enabled us to pool relevant datasets and employ an optimization routine to reconcile all available data on distributions from different types of sexual partnerships. Figure 1 and 2 reveal that in the past 6 months, the mean number of regular, casual and commercial sexual partners for an average Chinese MSM were 0.96 (0.59-1.18), 3.75 (1.72-6.25) and 1.61 (0.97-2.78) respectively, whose sum was comparable with the overall number of sexual partners 6.17 (3.45-9.96). Further, the mean number of sexual acts for these partnerships were 4.16 (2.87-5.63), 5.33 (2.84-7.28) and 2.38 (1.77-3.70) respectively in the same period, which corresponded to a total number of acts of 11.87 (8.87-15.25). Hence, an average Chinese MSM participates in approximately 4.33 (2.81-6.46), 1.42 (0.62-3.08), 1.48 (0.79-3.30) acts with each of his regular, casual and commercial partners in the past 6 months. Notably, all mean values represented the average behaviors of the entire MSM population. Different individuals would diverse sexual behavior, these were summarized by the distributions in Figure 1 and 2.

### Estimated annual HIV incidence

**Figure 4:**
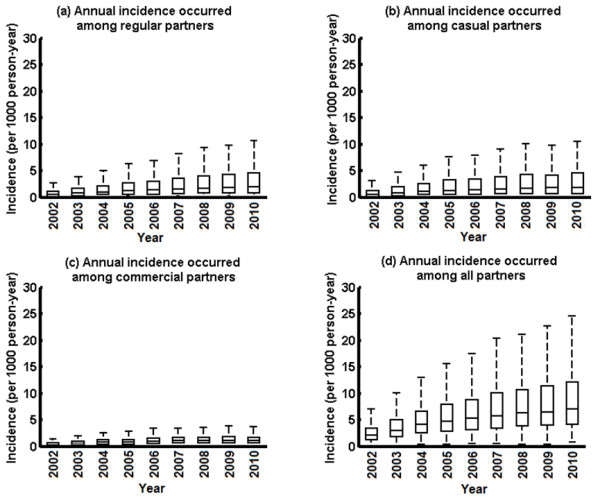
Annual HIV incidence occurred among (a) regular; (b) casual; (c) commercial; and (d) all partners among Chinese MSM during 2002–2010.

Our results showed that HIV incidences associated with all sexual partnership types followed an increasing trend during the period 2002–2010 (Figure 4). Mean annual HIV incidence among casual and commercial partners gradually increased from 0.55 (0.51-0.58) and 0.40 (0.39-0.42) per 1000 person-years (pys) in 2002 to 1.35 (1.28-1.42) and 1.00 (0.96-1.04) per 1000 pys in 2006 respectively, then both stabilized at relatively constant levels during the period 2006–2009. In comparison, mean annual incidence among regular MSM partners maintained a steady increasing trend from 0.47 (0.46-0.49) per 1000 pys in 2002 to 1.97 (1.90-2.06) per 1000 pys in 2010 (Figure 4). Accordingly, the overall median incidence increased 3.3-fold from 2.04 (1.96-2.12) to 7.02 (6.71-7.35) per 1000 pys during the same period. The percentage of susceptible Chinese MSM had a HIV incidence rate over 20 per 1000 yrs has sharply increased from 0.56% in 2002 to 6.38% in 2006 and 12.31% in 2010.

**Figure 5:**
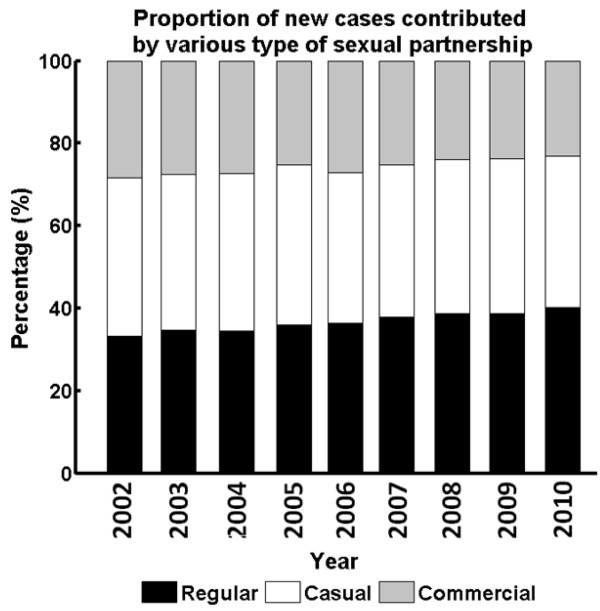
Composition of new HIV infections contributed by high-risk sexual Behaviors in various types of partnerships from 2002 to 2010.

Notably, in 2002, the proportion of new infections attributable to regular, casual and commercial partnerships were 34%, 37% and 29% respectively, with each partner type contributing approximately evenly. In comparison, the profile changed to 40%, 37% and 23% respectively in 2010. Infections attributable to regular partnerships started to dominate, with an overall increase of 6%, whereas the proportion attributable to commercial partnerships reduced by 6% (Figure 5).

## Discussion

In this study we presented, for the first time, a rigorous analysis on the distribution of number of sexual partners and acts among Chinese MSM. Here, we define, in a statistical sense, an ‘average’ Chinese MSM to be an individual who possesses behavioral characteristics that are exactly the means of the behavioral distributions. Notably, this differs from a ‘median’ subject as a mean value is always greater than a median estimate in lognormal distributions. We estimated that an average Chinese MSM has 0.96 (0.59‒1.18), 3.75 (1.72‒6.25) and 1.61 (0.97‒2.78) regular, casual and commercial partners respectively in the past 6 months. This confirms a previous finding that non-commercial casual partners represent the majority of MSM partnerships [19]. Consistently, the majority of sexual acts were casual (mean 5.33, 95% CI, 2.84‒7.28). This was similar to the number of regular acts 4.16 (95 CI%, 2.87‒5.63) but substantially higher than the number of commercial acts 2.38 (1.77‒3.70). However, in terms of sexual acts per partnership, our results suggest that casual and commercial acts were mostly once-off, and the frequency of sexual activities per regular partnership was as low as 4.33 (2.81-6.46) acts in the past 6 months. Our analysis also reported significant increases in condom usage among all types of MSM sexual partnerships. This is consistent with findings reported from other recent studies [10,20-22].

Sexual behaviors among Chinese MSM are more conservative in comparison with MSM in developed countries. In the United States, the median numbers of regular and casual partners of MSM in the past 12 months are reported to be 1 and 3 respectively [23], which are comparable to those in China (regular, 0.55 (0.34-0.95); casual 0.69 (0.31-1.22) in the past 6 months). In Australia, the average number of overall partnerships in the past 6 months have been reported to be 12–14 during the period 1999–2006 [24], almost two-fold the corresponding finding of 6.17 (3.45-9.96) in China. In addition, the average number of anal intercourse acts per regular partner in the past 6 months is 42–62 (1.6-2.4 acts/wk) in the US [24,25], whereas the number of acts per casual partnership is 26–52 (1–2 acts/wk [24-26]) in Australia. These are 5–10 times higher than our estimated number of acts among MSM in China. The more conservative sexual behaviours of Chinese MSM are likely a result of traditional familial values, societal expectations and self moral perception of homosexual acts among MSM in China [27-29].

We estimated a median HIV incidence of 7.02 (6.71-7.35) per 1000 pys among Chinese MSM in 2010. In contrast, the observed HIV incidence levels in some Chinese regions are 5–15 times higher (26-100/1000 pys [30-35]). The discrepancy is likely due to multiple reasons. All these empirical studies were performed in Chinese major cities where HIV prevalence among MSM was already known to be high [7,21,36]. In addition, MSM participants were selected mostly from MSM venues in these studies, where this MSM subgroup is more likely to participate in higher-risk sexual behaviors [37-40]. Our study collected a wide spectrum of studies from different geographical locations based on various methods of recruitment and sampling. Therefore, the estimated average incidence is likely a result of the average behaviors of overall MSM population in China. We found that a growing proportion of Chinese MSM have incidence rates exceeding 20 cases per 1000 pys (0.56% in 2002 vs. 12.31% in 2010). This subpopulation with relatively high incidence rates may coincide with the population sampled in some published studies [30-34].

Our analysis did not show significant temporal changes in the numbers of sexual partners nor acts for any MSM sexual types, but condom usage levels in all MSM sexual types significantly increased during the past decade. Hence, the counter-intuitive increase in estimated HIV incidence is likely due to the rapid increase of HIV prevalence among MSM. As HIV prevalence represents an accumulation of infected cases among the population, susceptible MSM are required to increase their condom usage substantially to avoid becoming infected. Our results suggest that despite significant increases in condom usage, the current levels of condom usage were not sufficient to offset the effects of elevated risk of infection as a result of increasing prevalence among the MSM population.

Our study is subject to several limitations. First, data for the distribution of the number of sexual acts are limited. There were only one available dataset for the distribution of commercial acts and two for overall sex acts, whereas data for regular and casual sex acts were absent. This substantially limited our analysis of the temporal trend in the number of sex acts. The majority of collected studies did not report the number of sex acts as one of their behavioral indicators. This reflects the knowledge gap between qualitative and quantitative HIV research in China, as only few studies were designed in a way that behavioral indicators can be quantified to estimate the risk of HIV transmission. Thus, we noted this important limitation in the empirical data, and restrained the interpretation of modeling results within the scope of the data and its representability of the behaviors of general MSM population in China. Second, the optimization process was performed based on an assumption of lognormal distributions of behavioral indicators. Although this was a valid assumption, the nature of lognormal distribution may lead to over-estimates of the actual numbers due to its skewness in large values. Besides, in a study setting, a participant was more likely to provide better account of partners and acts information for specific partnership types, but under-report the overall numbers from all partners. Hence, our optimization exercise may provide higher estimates than the actual empirical data. Third, we employed a linear regression model to estimate the temporal increase of condom usage. A linear assumption is valid when a short study period is considered. As condom usage levels have started to slowly plateau in recent years, the linear regression may over-estimate the actual usage rates in later years. Fourth, the current study did not take into consideration a number of factors with significant effects on sexual behaviors and HIV prevalence, including geographical differences, age differences and education levels. Recent studies have documented that HIV prevalence is much higher in Southwest China than any other parts of the country [4,36,41,42]. Whether this is an indication of more risky sexual behaviors among MSM in the Southwest remains unclear. Therefore, the limited number of studies included in our analysis may not be fully representable for China, although they were the best available studies. Also, younger MSM have been reported to be more sexually active [43] and MSM with lower education levels may be more prone to high-risk behaviors [44]. Due to limited information in our collected data, we were unable to stratify our analysis according to these factors.

Our study has important implications for HIV intervention strategies among MSM in China. Regular partnerships have become the main contributor of new HIV infections among Chinese MSM. This is consistent with the findings that the annual increase of condom usage among regular partnerships is lower than among casual and commercial partnerships. Regular partners tend to have more sexual acts per partnership and lower consistent condom usage. To date, there have been no specific harm reduction intervention programs targeting MSM in China. However, this is likely to change in the near future with increasing awareness from both the governmental and scientific communities. Regular partnerships should be the main focus and priority for any future interventions to curb the rapid transmission of HIV. Health resources should be directed towards intervention programs, such as HIV health education and voluntary counseling and testing (VCT), to encourage MSM and their regular sexual partners to be tested for HIV infection on a regular basis. Harm reduction programs that aim to increase condom usage should be implemented to improve safe sexual practices among Chinese MSM. These programs should target MSM venues, online chat-rooms and entertainment venues to promote condom usages not only with their casual and commercial partners, but also among regular partnerships.

## Conclusion

Conclusively, this study demonstrates an increase in HIV incidence among Chinese MSM of approximately 3.3-fold, from 2.04 (1.96-2.12) to 7.02 (6.71-7.35) per 1000 person-years during 2002-2010. The proportion of new infections attributed to regular partnerships increased from 34% to 40% during the same period. Regular partnerships are the main contributor of new HIV cases among MSM in China, public health intervention strategies are required to increase condom usage and HIV testing rates among regular partners to curb the growing trend HIV incidence.

## Competing interests

The authors declare that they have no competing interests.

## Authors' contributions

LZ was responsible for study design and data interpretation, writing and finalizing the manuscript and was the primary author of this manuscript. EPFC assisted with the study design, helped conduct the data collation and analysis and prepare the Methods and Results sections of the text. DPW contributed to data interpretation, supervision of the project and finalisation of manuscript. All authors read the final version of the manuscript.

## Pre-publication history

The pre-publication history for this paper can be accessed here:

http://www.biomedcentral.com/1471-2458/12/546/prepub

## Supplementary Material

Additional file 1 Supplementary materials. Click here for file
