# Supplementary material for: Distributions and trends in sexual behaviors and HIV incidence among men who have sex with men in China
Source: BMC Public Health. 2012 Jul 24;12:546. doi: 10.1186/1471-2458-12-546 (PMC3507867; doi:10.1186/1471-2458-12-546)
Supplement: Additional file 1 — Supplementary materials. [file 1471-2458-12-546-S1.doc]

**Supplementary Materials**

**Figure S1: Flow chart for selection of studies with number of articles (N).**

**
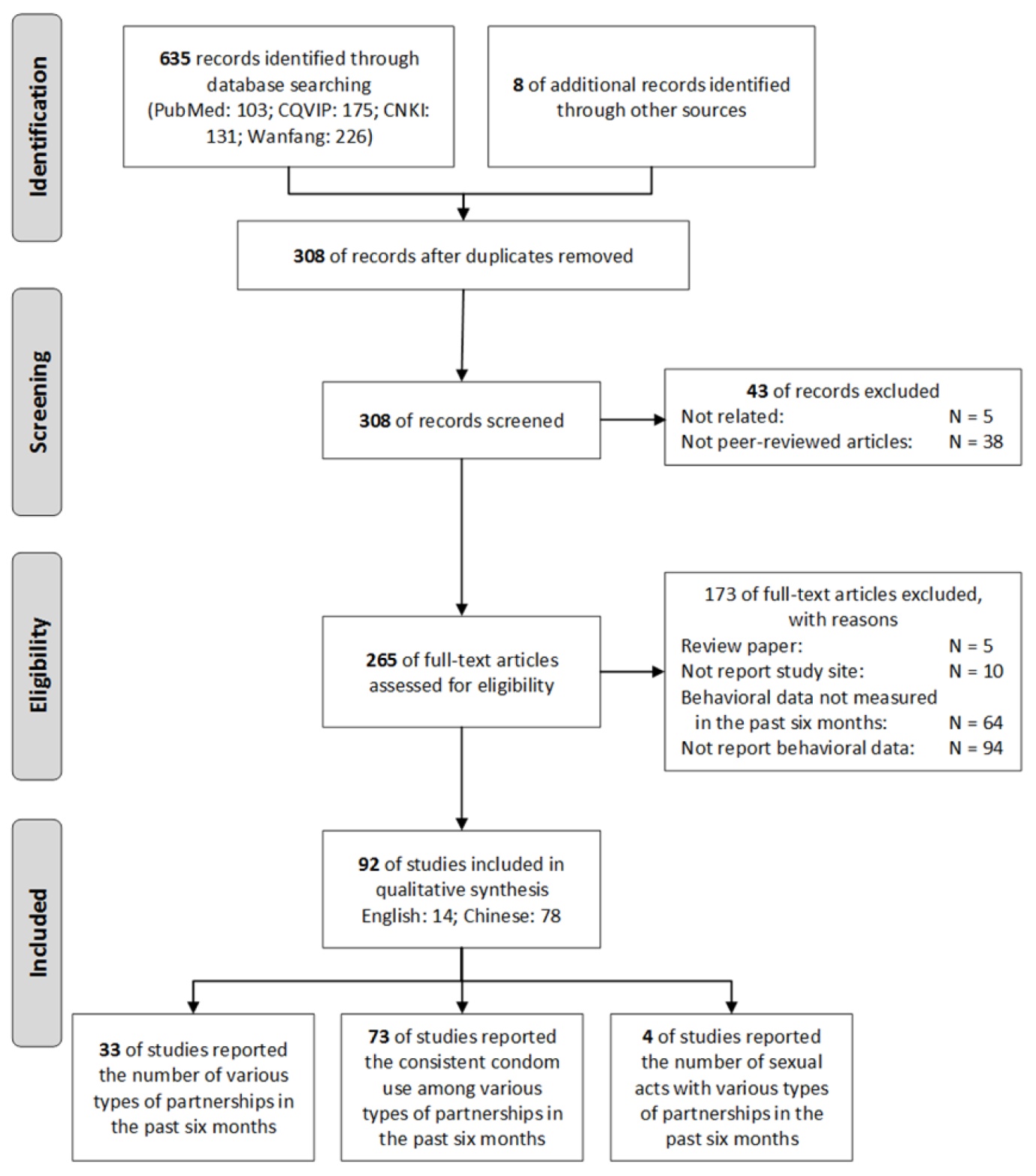
**

**Table S1**: Studies reporting number of male sexual partners among men who have sex with men in China

| **First author, published year** | **Study Design** | | | | | | **Number of different sexual partners in the past six months**  **Mean / Median*(Sampling size)** | | | |
| --- | --- | --- | --- | --- | --- | --- | --- | --- | --- | --- |
|  | **Study Period** | **Study location** | **Region** | **Age range (mean)** | **Method of recruitment** | **Method of sampling** | **Regular partners** | **Non-commercial casual partners** | **Commercial partners** | **Any male partners** |
| Choi KH, 2004 | 09/2001 – 01/2002 | Beijing | North | 18-69 (27) | MSM-identified bars, clubs, parks, and bathhouses | Convenience sampling. | N/A | N/A | N/A | Mean: 3.91  Median: 2.69  (n = 482) |
| Mao M, 2008 | 2003 | [Chengdu](http://maps.google.com.au/maps?hl=en&pq=成都+南充&xhr=t&q=成都地图&cp=3&client=firefox-a&rls=org.mozilla:en-GB:official&bav=on.2,or.r_gc.r_pw.&biw=1512&bih=746&bs=1&um=1&ie=UTF-8&hq=&hnear=0x36efc52300447721:0xb98652ce2e240e02,Chengdu,+Sichuan,+China&gl=au&ei=snbfTfagI4-mvQOJtLHcBQ&sa=X&oi=geocode_result&ct=title&resnum=1&sqi=2&ved=0CCEQ8gEwAA) & Nanchong, Sichuan | Southwest | 16-76 (28.1) | Gay bars, hotlines, websites, parks | N/A | N/A | N/A | N/A | Mean: 5.36  Median: 2.31  (n = 579) |
| He Q, 2005 | 04/2003 – 05/2003 | Guangzhou, Guangdong | South Central | 18-55 | Gay-oriented activity venue, Internet | Peer-referral, Snowball sampling, gay website advertisement | Mean: 0.49  Median: 0.25  (n = 121) | N/A | N/A | N/A |
| Yang HT, 2006 | 05/2004 – 07/2004 | Jiangsu | East | 18-56 (28.0) | Gay-oriented activity venue | N/A | Mean: 1.15  Median: 1.05  (n = 92) | N/A | N/A | Mean: 6.54  Median: 2.98  (n = 222) |
| Ma X, 2007 | 2004 | Beijing | North | ≥18 | MSM community | Respond Driven Sampling | N/A | N/A | N/A | Mean: 3.13  Median: 1.67  (n = 325) |
| Li X, 2006 | 2004 | Xi’an | Northwest | N/A (28.0) | Gay-oriented activity venue | N/A | N/A | N/A | N/A | Mean: 4.87  Median: 2.09  (n = 230) |
| Zhang BC, 2007 | 2004 | 6 cities (Chongqing, Shenyang, Dalian, Qingdao, Nanjing, Xi’an) | N/A | 15-72 (27.6) | Gay bars and volunteer activity venue | Snowball sampling | N/A | N/A | Mean: 3.36  Median: 1.93  (n = 117) | Mean: 4.33  Median: 1.93  (n = 1123) |
| Choi KH, 2007 | 09/2004 – 06/2005 | Shanghai | East | 18-56 (28.0) | MSM venues | Respond Driven Sampling | N/A | N/A | N/A | Mean: 2.97  Median: 1.39  (n = 477) |
| Xu J, 2007 | 03/2005 – 12/2005 | Hefei, Anhui | East | 18-42 (23.3) | N/A | Respond Driven Sampling, peer-referral, gay website advertisement | N/A | Mean: 0.77  Median: 0.72  (n = 223) | N/A | Mean: 0.99  Median: 0.95  (n = 223) |
| Li X, 2008 | 06/2005 – 11/2005 | Beijing | North | 17-54 | Website advertisement, outreach in MSM clubs, bars, parks and bathhouses | N/A | N/A | N/A | N/A | Mean: 4.61  Median: 1.81  (n = 526) |
| Zhu JL, 2007 | 06/2005 – 12/2005 | Hefei, Anhui | East | 18-29 (20.4) | Gay websites | Respond Driven Sampling, peer-referral | N/A | N/A | N/A | Mean: 1.49  Median: 0.96  (n = 122) |
| Zhang BC, 2008 | 2005-2006 | 9 cities (Shanghai, Nanjing, Harbin, Shenyang, Wuhan, Zhengzhou, Chongqing, Chengdu, Xi’an) | N/A | 13-78 (29.1) | Gay-oriented activity venue | Snowball sampling | N/A | Mean: 4.42  Median: 1.95  (n = 650) | N/A | Mean: 6.60  Median: 2.57  (n = 2250) |
| Sun ZX, 2007 | 2006 | Zhejiang | East | ≥15 | Participants in a gay-oriented activity | Gay website advertisement | N/A | N/A | N/A | Mean: 1.77  Median: 1.99  (n = 43) |
| Choi J, 2007 | 2006 | Nanchang, Jiangxi | East | 16-50 (25.3) | Gay bars | N/A | N/A | N/A | N/A | Mean: 1.28  Median: 0.67  (n = 99) |
| Ma X, 2007 | 2006 | Beijing | North | ≥16 | MSM community | Respond Driven Sampling | N/A | N/A | N/A | Mean: 6.25  Median: 3.09  (n = 540) |
| Li N, 2007 | 2006 | Henan | South Central | 17-68 (28.3) | National HIV/AIDS Sentinel Surveillance | N/A | N/A | N/A | N/A | Mean: 4.72  Median: 2.56  (n = 160) |
| Fu LJ, 2007 | 02/2006 – 12/2006 | Shaoxing, Zhejiang | East | 19-41 (26.0) | Peer-referral | Snowball sampling | N/A | N/A | N/A | Mean: 1.28  Median: 0.72  (n = 55) |
| Ma J, 2007 | 04/2006 – 10/2006 | Tianjin | North | N/A (25.2) | Gay websites | N/A | *STD group:* Mean: 1.38  Median: 1.02  (n = 99)  *Non-STD group:*  Mean: 1.21  Median: 0.49  (n = 334) | N/A | N/A | *STD group*: Mean: 4.66  Median: 2.98  (n = 99)  *Non-STD group*:  Mean: 2.36  Median: 1.23  (n = 334) |
| Zhang D, 2007 | 05/2006 – 08/2006 | N/A | N/A | ≥18 | Gay websites | N/A | N/A | N/A | N/A | Mean: 2.72  Median: 1.29  (n = 2364) |
| Ouyang L, 2008 | 06/2006 – 09/2006 | Chongqing | Southwest | 16-60 (30.2) | MSM bars, bathhouse | Snowball sampling | N/A | N/A | N/A | Mean: 2.73  Median: 1.72  (n = 519) |
| Chen SH, 2008 | 07/2006 – 08/2006 | Nanning, Guangxi | South Central | 18-45 (26.2) | N/A | Peer-referral, website advertisement | N/A | N/A | N/A | Mean: 1.52  Median: 0.33  (n = 185) |
| Feng L, 2009 | 07/2006 – 09/2006 | Chongqing | Southwest | N/A | MSM activity venues (clubs, bars, bathhouses, outdoor cruising areas), community outreach, peer-recruitment and web-based recruitment | Venue-based and cruising  area–based convenience sampling, | N/A | N/A | N/A | Mean: 2.63  Median: 1.19  (n = 1000) |
|  | 07/2007 – 09/2007 | Ibid | Ibid | Ibid | Ibid | Ibid | N/A | N/A | N/A | Mean: 3.28  Median: 1.10  (n = 1044) |
| Zhou J, 2008 | 09/2006 – 12/2006 | Guiyang, Guizhou | Southwest | 15-49 (24.0) | Gay venues, websites, hotlines | N/A | N/A | N/A | N/A | Mean: 1.34  Median: 0.88 |
| Liu H, 2009 | 2007 | Shenzhen, Guangdong | South Central | 18-45 | MSM venues (sauna, bar, public park) | Respondent-Driven Sampling | N/A | N/A | N/A | Mean: 3.19  Median: 1.65  (n = 293) |
| Zhou SJ, 2008 | 2007 | Chongqing | Southwest | ≥16 | Gay bathrooms, bars, activity centers | N/A | N/A | N/A | N/A | Mean: 3.10  Median: 1.95  (n = 339) |
| Wang Y, 2008 | 01/2007 | Mianyang, Sichuan | Southwest | 16-57 (24.8) | MSM community | Respond Driven Sampling | Mean: 0.78  Median: 0.48  (n = 201) | Mean: 1.52  Median: 0.20  (n = 201) | Mean: 0.97  Median: 0.73  (n = 200) | Mean: 1.34  Median: 0.55  (n = 201) |
| Feng Y, 2010 | 03/2007 – 06/2007 | Chengdu, Sichuan | Southwest | 16.8-44.5 | MSM community | Snowball sampling | N/A | Mean: 2.72  Median: 1.27  (n = 512) | N/A | N/A |
| Zhao HP, 2009 | 01/2008 – 10/2008 | Harbin, Heilongjiang | Northeast | 17-52 (25.0) | MSM who came to voluntary counseling and testing clinics | N/A | N/A | N/A | N/A | Mean: 4.46  Median: 1.96  (n = 89) |
| Feng LG, 2010 | 02/2008 – 06/2008 | Chongqing | Southwest | N/A | Website, gar bars, clubs, parks, bathhouse | Snowball sampling | N/A | N/A | N/A | Mean: 2.19  Median: 1.11  (n = 945) |
| Xu J, 2010 | 03/2008 – 07/2008 | Beijing, Harbin, Zhengzhou, Chengdu | N/A | N/A (29.2) | N/A | Snowball sampling | N/A | N/A | N/A | Mean: 2.90  Median: 1.81  (n = 1677) |
| Liang L, 2009 | 04/2008 – 06/2008 | N/A | N/A | N/A | MSM who had voluntary counselling and testing services | Snowball sampling | N/A | N/A | N/A | Mean: 2.32  Median: 1.53  (n = 450) |
| Wang ZJ, 2010 | 05/2008 – 06/2009 | Yangzhou, Jiangsu | East | 18-78 (33.5) | Website advertisement | N/A | N/A | N/A | N/A | Mean: 3.28  Median: 1.10  (n = 750) |
| Wei C, 2011 | 2008 | Jinan, Shandong | East | N/A | MSM volunteer group | Respondent-driven sampling | N/A | N/A | N/A | Mean: 2.73  Median: 1.62  (n = 402) |
| Xu J, 2011 | 04/2008 – 01/2009 | Liaoning | Northeast |  | MSM community | N/A | N/A | N/A | N/A | Mean: 1.64  Median: 0.81  (n = 436) |
| * Spearman correlation tests were performed to investigate the associations between median/mean of the number of sexual partners and the year of study for each sexual type. For studies spanned more than one year, the mid-point of the time interval was used. The correlation results are: regular partnership, median vs. year (*p* = 0.95, *r* = 0.05), mean vs. year (*p* = 0. 52, *r* = 0.36); : non-commercial casual partnership, median vs. year (*p* = 0.92, *r* = -0.21), mean vs. year (*p* = 0.75, *r* = 0.32); commercial partnership: insufficient data for correlation tests; overall partnership, median vs. year (*p* = 0.12, *r* = -0.30), mean vs. year (*p* = 0.20, *r* = -0.25). | | | | | | | | | | |

**Table S2**: Studies reporting number of acts with male partners among men who have sex with men in China

| **First author, published year** | **Study Design** | | | | | | **Number of acts with different types of partnerships in past six months**  **Mean / Median*** | | | |
| --- | --- | --- | --- | --- | --- | --- | --- | --- | --- | --- |
|  | **Study Period** | **Study location** | **Region** | **Age range (mean)** | **Method of recruitment** | **Method of sampling** | **Regular partners** | **Non-commercial casual partners** | **Commercial partners** | **Any male partners** |
| Cai WD, 2005 | 08/2004 – 11/2004 | Shenzhen | South Central | 17-58 (25.9) | Gay bars, fitness centers | N/A | N/A | N/A | N/A | Mean: 11.02  Median: 3.65  (n = 268) |
| Yang HT, 2006 | 05/2004 – 07/2004 | Jiangsu | East | 18-56 (28.0) | Gay-oriented activity venue | N/A | N/A | N/A | Mean: 2.38  Median: 1.51  (n = 30) | N/A |
| Zhang X, 2007 | 01/2005 – 12/2006 | Beijing | North | 18-55 (26.1) | VCT clinic at Chaoyang District CDC | N/A | N/A | N/A | N/A | *Insertive:*  Mean: 4.90  Median: 4.81  *Receptive:*  Mean: 5.02  Median: 4.77  (n = 753) |
| Xiao Y, 2009 | 07/2006 – 09/2007 | Chongqing | Southwest | 18-68 (27.7) | Gay-oriented volunteer workgroups, gay bars, bathhouses, clubs, saunas, gay websites | Multiplier methods: venue-based recruitment, Internet  advertisements, community outreach, and snowball sampling | N/A | N/A | Mean: 1.45  Median: 1.29  (n = 69) | N/A |
| * There is insufficient data to perform Spearman correlation for median/mean number of sexual acts and the year of study. | | | | | | | | | | |

**Table S3: Studies reporting the consistent condom use in the** past 6 months among men who have sex with men with different types of male sexual partners in China

| **First author, published year** | **Study Design** | | | | | | **Consistent condom use with different types of partnerships**  **in the past six months, n/N (%)** | | | |
| --- | --- | --- | --- | --- | --- | --- | --- | --- | --- | --- |
|  | **Study Period** | **Study location** | **Region** | **Age range (mean)** | **Method of recruitment** | **Method of sampling** | **Regular partners** | **Non-commercial casual partners** | **Commercial partners** | **Any male partners** |
| Lan Y, 2004 | 09/2003 – 12/2003 | Sichuan | Southwest | N/A (28.1) | MSM venues (bars, bathhouses, hotlines, websites, parks) | Snowball sampling | 53/336 (15.8%) | 53/325 (16.3%) | 32/99 (32.3%) | N/A |
| Li X, 2009 | 2003 | Xi’an, Shaanxi | Northwest | 18-55 (30.2) | HIV National Sentient Surveillance Site | N/A | N/A | N/A | N/A | 9/78 (17.0%) |
|  | 2004 | Ibid | Ibid | 17-61 (28.0) | Ibid | N/A | N/A | N/A | N/A | 56/258 (24.7%) |
|  | 2005 | Ibid | Ibid | 18-62 (27.6) | Ibid | N/A | N/A | N/A | N/A | 27/106 (43.6%) |
|  | 2006 | Ibid | Ibid | 16-62 (27.0) | Ibid | N/A | N/A | N/A | N/A | 121/393 (35.1%) |
|  | 2007 | Ibid | Ibid | 18-64 (27.5) | Ibid | N/A | N/A | N/A | N/A | 81/256 (41.1%) |
|  | 2008 | Ibid | Ibid | 16-68 (29.3) | Ibid | N/A | N/A | N/A | N/A | 120/400 (33.7%) |
| Jiang SM, 2006 | 2004 | Weihai, Shandong | East | 19-59 (29.7) | N/A | N/A | N/A | N/A | N/A | 19/59 (32.2%) |
| He Q, 2005 | 04/2004 – 05/2004 | Guangzhou, Guangdong | South Central | 18-55 | Gay-oriented activity venue, Internet | Peer-referral, Snowball sampling, gay website advertisement | N/A | N/A | N/A | 33/91 (36.3%) |
| Yang HT, 2006 | 05/2004 – 07/2004 | Jiangsu | East | 18-56 (28.0) | Gay-oriented activity venue | N/A | N/A | N/A | 17/28 (60.7%) | 61/175 (34.9%) |
| Zhang D, 2007 | 2002 | Harbin, Heilongjiang | Northeast | 18-67 | Gay-identified venues | N/A | N/A | N/A | N/A | 28/170 (16.5%) |
|  | 2004 | Ibid | Ibid | 18-75 | Ibid | N/A | N/A | N/A | N/A | 56/265 (21.1%) |
|  | 2006 | Ibid | Ibid | 18-69 | Ibid | N/A | N/A | N/A | N/A | 155/425 (36.5%) |
| Tao XY, 2006 | 03/2004 – 10/2004 | Shenzhen, Guangdong | South Central | 17-47 (24.0) | Gay bars | N/A | N/A | N/A | N/A | 36/114 (31.6%) |
| He Q, 2008 | 2004 | Chengdu, Sichuan | Southwest | 17-62 (26.1) | MSM community | Snowball sampling | N/A | N/A | 10/44 (22.7%) | 55/170 (32.4%) |
|  | 2005 | Ibid | Ibid | 17-49 (25.4) | Ibid | Ibid | N/A | N/A | 24/41 (58.5%) | 81/184 (44.0%) |
|  | 2006 | Ibid | Ibid | 16-58 (25.8) | Ibid | Ibid | N/A | N/A | 17/36 (47.1%) | 86/207 (41.5%) |
|  | 2007 | Ibid | Ibid | 16-74 (27.7) | Ibid | Ibid | N/A | N/A | 19/34 (55.9%) | 85/197 (43.1%) |
| Tian XB, 2006 | 06/2005 – 09/2005 | Nanchong, Sichuan | Southwest | 16-65 | Gay-identified venues, hotlines, websites | Snowball and convenience sampling | 4/84 (4.8%) | 0.9% (1/109) | 3/40 (7.5%) | N/A |
| Xing JM, 2007 | 10/2005 – 12/2005 | Changsha, Hunan | South Central | 14-63 (29.8) | Gay bars, bath house, public toilet | N/A | N/A | N/A | 18/36 (50.0%) | N/A |
| Chen SC, 2007 | 07/2005 – 09/2005 | Hangzhou, Zhejiang | East | 18-70 (28.0) | Gay-identified venues, websites | Snowball sampling | N/A | N/A | 4/17 (23.5%) | 118/259 (45.6%) |
| Hu Q, 2006 | 08/2005 | Nanchang, Jiangxi | East | 15-60 (25.0) | Websites, peer referral, gay bars, clubs | N/A | N/A | N/A | N/A | 33/144 (22.9%) |
| Liao MZ, 2006 | 09/2005 – 11/2005 | Shandong | East | 18-73 (26.0) | Gay-identified venues | N/A | N/A | N/A | N/A | 32/109 (29.4%) |
| Lai YH, 2006 | 2004 – 2005 | Shenzhen, Guangdong | South Central | 15-57 (26.0) | Gay-identified venues | N/A | N/A | N/A | N/A | 84/203 (41.4%) |
| Lu CG, 2006 | 12/2003 – 11/2005 | Guiyang, Guizhou | Southwest | 15-81 | VCT clinic | Snowball sampling | N/A | N/A | N/A | 19/170 (11.2%) |
| Lau JT, 2006 | 02/2004 – 08/2005 | Kunming, Yunnan | Southwest | 15-75 | Gay-identified venues, website | Multiple sampling (Convenience, snowball) | N/A | 331/730 (45.3%) | 90/130 (69.2%) | N/A |
| Zhang CQ, 2006 | 2005 | Jinan, Shandong | East | N/A | Peer-referral | Snowball sampling | N/A | N/A | N/A | 13/47 (27.7%) |
| Zhu YW, 2007 | 03/2006 – 07/2006 | Jinan, Shandong | East | 17-66 (25.4) | Jinan CDC | N/A | N/A | N/A | N/A | 116/375 (30.9%) |
| Zhang D, 2007 | 05/2006 – 08/2006 | N/A | N/A | ≥18 | Gay websites | N/A | N/A | N/A | N/A | 406/1375 (29.5%) |
| Cai CF, 2008 | 06/2006 – 12/2006 | Zhejiang | East | 16-46 | Gay-identified venues | N/A | N/A | N/A | 12/18 (66.7%) | 52/103 (50.5%) |
| Quyang L, 2008 | 06/2006 – 09/2006 | Chongqing | Southwest | 16-60 (30.2) | MSM bars, bathhouse | Snowball sampling | N/A | N/A | N/A | 65/284 (22.9%) |
| Wang L, 2007 | 06/2006 – 07/2006 | N/A | N/A | 18-69 (30.3) | Gay-identified venues | N/A | N/A | N/A | N/A | 145/407 (35.6%) |
| Cai X, 2007 | 07/2006 – 12/2006 | Liaocheng, Shandong | East | 18-38 (26.0) | Health Forum | N/A | N/A | N/A | N/A | 35/201 (17.4%) |
| Chen S, 2007 | 07/2006 – 08/2006 | Nanning, Guangxi | South Central | 18-45 (26.2) | Website advertisement, peer-referral | N/A | N/A | N/A | N/A | 55/156 (35.3%) |
| Wu J, 2008 | 10/2006 – 12/2006 | Shanghai | East | 18-54 (26.6) | Gay-identified venues | N/A | 18/80 (22.5%) | 71/144 (49.3%) | N/A | N/A |
| Wang CH, 2007 | 06/2003 – 10/2006 | Chengde, Hebei | North | 15-28 (21.5) | Gay-identified venues | Snowball sampling | N/A | N/A | N/A | 25/82 (30.5%) |
| Chen SH, 2007 | 06/2006 | Guangxi | South Central | N/A (27.7) | Website | N/A | N/A | N/A | N/A | 141/548 (25.7%) |
| Li Y, 2007 | 08/2006 – 09/2006 | Lanzhou, Gansu | Northwest | 18-61 (30.0) | Gay-identified venues | Snowball sampling | N/A | N/A | N/A | 106/202 (52.5%) |
| Wang HL, 2008 | 08/2006 – 10/2006 | Shenyang, Liaoning | Northeast | 18-60 | N/A | N/A | 69/212 (32.5%) | 98/218 (45.0%) | 95/188 (50.5%) | 100/200 (50.0%) |
| Zeng G, 2009 | 09/2006 | 18 cities in Heilongjiang, Jilin, Liaoning, Inner Mongolia, Ningxia, Gansu, Chongqing | N/A | N/A | Gay-identified venues | Snowball sampling and respond Driven Sampling | N/A | N/A | N/A | 1163/4118 (28.2%) |
|  | 05/2008 | Ibid | N/A | N/A | Ibid | Ibid | N/A | N/A | N/A | 2114/4753 (44.5%) |
| Zhou J, 2008 | 09/2006 – 12/2006 | Guiyang, Guizhou | Southwest | 15-49 (24.0) | Gay venues, websites, hotlines | N/A | N/A | N/A | N/A | 70/255 (27.5%) |
| Li N, 2007 | 2006 | Henan | South Central | 17-68 (28.3) | National HIV/AIDS Sentinel Surveillance | N/A | N/A | N/A | N/A | 41/147 (27.9%) |
| Sun ZX, 2007 | 2006 | Zhejiang | East | ≥15 | Participants in a gay-oriented activity | Gay website advertisement | N/A | N/A | N/A | 22/68 (32.4%) |
| Choi J, 2007 | 2006 | Nanchang, Jiangxi | East | 16-50 (25.3) | Gay bars | N/A | N/A | N/A | N/A | 38/58 (65.5%) |
| Duan YW, 2010 | 12/2006 – 02/2007 | Mianyang, Sichuan | Southwest | ≥18 | Peer-referral | N/A | 34/137 (24.6%) | 23/75 (30.8%) | 13/33 (39.3%) | N/A |
|  | Ibid | Yibin, Sichuan | Ibid | Ibid | Ibid | N/A | 43/142 (30.3%) | 33/77 (43.2%) | 23/58 (40.4%) | N/A |
| China Global Fund AIDS Program Round 5, 2007 | 2006 | Chongqing | Southwest | 18-65 | MSM venues | Snowball sampling | N/A | N/A | 27/44 (61.4%) | 250/786 (31.8%) |
|  | 2007 | Ibid | Ibid | 18-65 | Ibid | Ibid | N/A | N/A | 17/30 (56.7%) | 271/647 (41.9%) |
| Liu LY, 2008 | 2006 | Mudanjiang, Heilongjiang | Northeast | N/A (29.0) | Gay-identified venues | N/A | N/A | 55/202 (27.2%) | 144/202 (71.5%) | N/A |
| Chen SH, 2010 | 2006 | Nanning, Guangxi | South Central | 14-68 (26.2) | Heath check at Nanning CDC | Convenience and snowball sampling | N/A | N/A | N/A | 55/156 (35.3%) |
|  | 2007 | Ibid | Ibid | 14-68 (25.5) | Ibid | Ibid | N/A | N/A | N/A | 83/196 (42.3%) |
|  | 01/2008 – 06/2008 | Ibid | Ibid | 14-68 (27.4) | Ibid | Ibid | N/A | N/A | N/A | 140/402 (34.8%) |
|  | 07/2008 – 12/2008 | Ibid | Ibid | 14-68 (27.3) | Ibid | Ibid | N/A | N/A | N/A | 169/375 (45.1%) |
| Xiao Y, 2009 | 07/2006 -09/2007 | Chongqing | Southwest | 18-68 (27.7) | Gay-oriented volunteer workgroups, gay bars, bathhouses, clubs, saunas, gay websites | Multiplier methods: venue-based recruitment, Internet  advertisements, community outreach, and snowball sampling | N/A | N/A | 57/71 (80.3%) | 503/1376 (36.6%) |
| Feng Y, 2010 | 03/2007 – 06/2007 | Chengdu, Sichuan | Southwest | 16.8-44.5 | MSM community | Snowball sampling | 84//358 (23.5%) | 149/386 (38.6%) | N/A | N/A |
| Wang Y, 2008 | 12/2006 – 01/2007 | Mianyang, Sichuan | Southwest | 16-57 (24.8) | MSM network | Respond Driven Sampling | 34/137 (24.8%) | 23/76 (30.3%) | 13/33 (39.4%) | 63/201 (31.3%) |
| Xu ZH, 2009 | 04/2007 – 09/2007 | Tieling, Liaoning | Northeast | 16-45 | Gay-identified venues | N/A | N/A | N/A | N/A | 12/65 (18.5%) |
| Xu YF, 2008 | 08/2007 – 09/2007 | Nanning, Guangxi | South Central | 17-38 | Website advertisement, peer-referral, gay bar activity advertisement | N/A | N/A | N/A | N/A | 99/230 (43.0%) |
| Guo Y, 2009 | 06/2007 – 10/2007 | Tianjin | North | 17-57 (27.9) | Gay bars | N/A | N/A | N/A | N/A | 63/133 (47.4%) |
| Zou H, 2010 | 06/2007 – 08/2007 | Beijing and Urumqi | North and Northwest | 18-56 | Gay websites | N/A | N/A | N/A | N/A | 110/281 (39.1%) |
| Zeng H, 2011 | 07/2007 – 08/2007 | Chongqing | Southwest | 15-65 (26.4) | Website, MSM venues | Snowball sampling | N/A | N/A | 38/50 (76.0%) | 271/647 (41.9%) |
| Cai YM, 2009 | 10/2007 – 12/2007 | Shenzhen, Guangdong | South Central | 18-45 | Gay-identified venues | Respondent-Driven Sampling | N/A | N/A | 25/33 (75.8%) | 184/323 (57.0%) |
| Guo LY, 2008 | 2007 | Jining, Shandong | East | 18-50 | Gay activity venues, website (Rainbow Online) | N/A | N/A | N/A | N/A | 20/106 (18.9%) |
| Weng YQ, 2009 | 2007 | Guangxi | South Central | 18-57 (27.9) | MSM network, MSM venues | Snowball sampling | N/A | N/A | N/A | 63/239 (26.4%) |
| Zhou SJ, 2008 | 2007 | Chongqing | Southwest | ≥16 | Gay bathrooms, bars, activity centers | N/A | N/A | N/A | N/A | 86/350 (24.6%) |
| Liu H, 2009 | 2007 | Shenzhen, Guangdong | South Central | 18-45 | MSM venues (sauna, bar, public park) | Respondent-Driven Sampling | N/A | N/A | 21/26 (80.8%) | 167/293 (57.0%) |
| Guo W, 2008 | 09/2007 – 01/2008 | Langfang, Hebei | North | 18-54 | Gay-identified venues | N/A | N/A | N/A | N/A | 81/174 (46.6%) |
| Sun ML, 2009 | 04/2008 – 07/2008 | Dalian, Liaoning | Northeast | N/A | MSM venues | N/A | N/A | N/A | N/A | 172/348 (49.4%) |
| Zhao HP, 2009 | 01/2008 – 10/2008 | Harbin, Heilongjiang | Northeast | 17-52 (25.0) | MSM who came to voluntary counseling and testing clinics | N/A | N/A | N/A | N/A | 38/89 (42.7%) |
| Ding XB, 2010 | 02/2008 – 06/2008 | Chongqing | Southwest | 18-67 (26.3) | MSM network | Snowball sampling | N/A | N/A | N/A | 231/629 (36.7%) |
| Liang L, 2009 | 04/2008 – 06/2008 | N/A | N/A | N/A | MSM who participated in voluntary counseling and testing services | Snowball sampling | N/A | N/A | N/A | 176/429 (41.0%) |
| Wen XQ, 2010 | 04/2008 – 06/2008 | Guilin, Guangxi | South Central | N/A | MSM venues | Snowball sampling | N/A | N/A | 4/13 (30.8%) | 31/205 (15.1%) |
| Miao ZF, 2009 | 04/2008 – 06/2008 | Yinchuan, Ningxia | Northwest | 18-55 (27.7) | MSM venues | Snowball sampling | N/A | N/A | N/A | 107/191 (56.0%) |
| Chen SH, 2010 | 04/2008 – 06/2008 | Nanning, Guangxi | South Central | 16-68 (27.4) | Health check at CDC | Snowball sampling | N/A | N/A | N/A | 140/402 (34.8%) |
| Han DL, 2009 | 04/2008 – 08/2008 | Chengdu, Sichuan | Southwest | ≥18 | MSM venues | Snowball sampling | N/A | N/A | N/A | 178/467 (38.1%) |
| Shi WD, 2009 | 05/2008 – 08/2008 | Wuhan, Hubei | North | 18-60 (28.3) | VCT clinics | N/A | N/A | N/A | N/A | 176/379 (46.4%) |
| Zhang M, 2009 | 05/2008 – 06/2008 | Ürümqi, Xinjiang | Northwest | 18-54 (27.4) | Peer-referral | Respondent-Driven Sampling | N/A | N/A | N/A | 69/189 (35.5%) |
| Qu L, 2009 | 10/2008 – 12/2008 | Inner Mongolia | North | 18-63 (27.0) | MSM venues | N/A | N/A | N/A | N/A | 261/598 (43.6%) |
| Wang XL, 2009 | 11/2008 – 12/2008 | Tangshan, Hebei | North | 19-53 | MSM venues | N/A | N/A | N/A | N/A | 9/49 (18.4%) |
| Wang M, 2009 | 2008 | Kunming, Yunnan | Southwest | 16-80 | MSM venues, websites | Radom sampling | N/A | 192/306 (62.7%) | N/A | N/A |
| Wei C, 2011 | 2008 | Jinan, Shandong | East | N/A | MSM volunteer group | Respondent-driven sampling | N/A | N/A | N/A | 85/304 (28.0%) |
| Xu JJ, 2011 | 04/2008 – 01/2009 | Liaoning | Northeast | ≥18 | MSM community | N/A | 180/371 (48.5%) | 254/436 (58.3%) | N/A | N/A |
| Ma YG, 2010 | 04/2008 – 06/2009 | Tibet | Southwest | N/A | MSM who participated in voluntary counseling and testing services | N/A | N/A | N/A | N/A | 7/14 (50.0%) |
| Wang ZJ, 2010 | 05/2008 – 06/2009 | Yangzhou, Jiangsu | East | 18-78 (33.5) | Website advertisement | N/A | N/A | N/A | N/A | 269/544 (49.5%) |
| Gong CT, 2010 | 10/2008 – 06/2009 | Quanzhou, Fujian | East | 19-46 (22.0) | MSM venues | Snowball sampling | N/A | N/A | 2/5 (40%) | 78/145 (53.8%) |
| Ji GP, 2010 | 2008 1^st^ round | Anhui | East | 18-72 (26.7) | N/A | N/A | N/A | N/A | 11/23 (47.8%) | 148/374 (39.6%) |
|  | 2008 2^nd^ round | Ibid | Ibid | 18-72 (24.8) | N/A | N/A | N/A | N/A | 11/21 (52.4%) | 126/378 (33.3%) |
|  | 2009 | Ibid | Ibid | 18-72 (26.0) | N/A | N/A | N/A | N/A | 21/35 (60.0%) | 243/565 (43.0%) |
| Shi JC, 2010 | 04/2009 – 11/2009 | Nanyang, Henan | South Central | 17-66 (32.0) | Websites, peer-referral, MSM activities | N/A | N/A | N/A | N/A | 31/132 (23.5%) |
| Wang T, 2010 | 2009 | Zhongshan, Guangdong | South Central | 17-52 (26.0) | MSM group activities and voluntary counseling and testing clinics | N/A | N/A | N/A | N/A | 56/163 (34.4%) |
| Zhang JM, 2010 | 03/2010 – 07/2010 | Jianshui, Yunnan | Southwest | 18-61 | N/A | Snowball sampling | N/A | N/A | N/A | 27/110 (24.5%) |

* Linear regressions were used to investigate the temporal trend of condom usage during 2002-2010 for each sexual type. For studies spanned more than one year, the mid-point of the time interval was used. The slopes of the linear regressions are: 3.58% (2.98-4.12%), 5.55 (4.55-6.54), 5.03% (4.19-5.74%) for regular, casual and commercial partners respectively. All slopes are significantly greater than zero.

**References**

1. Choi KH, Gibson DR, Han L, Guo Y: **High levels of unprotected sex with men and women among men who have sex with men: a potential bridge of HIV transmission in Beijing, China**. *AIDS Educ Prev* 2004, **16**(1):19-30.

2. Mao M, Lan YJ, Zhou DL, Gu Y, Zhang JX, Xie YQ, Niu J: **[Frequency of Condom Use in Anal Copulation Among Homosexual Men]**. *J Prev Med Inf* 2008, **24**(7):493-495.

3. He Q, Wang Y, Lin P, Zhang Z-b, Zhao X-x, Xu H-f: **[KAP study on AIDS among men who have sex with men in Guangzhou, Guangdong province]**. *Chinese Journal of Disease Control & Prevention* 2005, **9**(2):106-108.

4. Yang HT, Ding JP, Chen GH, Li XF, Zhang BC, Guan WH: **[Behavioral features of Men Who Have Sex with Men in Jiangsu]**. *Jiangsu Prev Med* 2006, **17**(1):1-4.

5. Ma X, Zhang Q, He X, Sun W, Yue H, Chen S, Raymond HF, Li Y, Xu M, Du H *et al*: **Trends in Prevalence of HIV, Syphilis, Hepatitis C, Hepatitis B, and Sexual Risk Behavior Among Men Who Have Sex With Men. Results of 3 consecutive respondent-driven sampling surveys in Beijing, 2004 through 2006**. *J Acquir Immune Defic Syndr* 2007, **45**(5):581-587.

6. Li X, Wang BS, Li XF, Cui M, Zhang BC, Chang WH, Xing AH: **[Survey on HIV/AIDS-related high-risk behaviors among men who have sex with men in Xi’an]**. *Chin J Public Health* 2006, **22**(5):520-521.

7. Zhang BC, Zeng Y, Xu H, Li XF, Zhou SJ, Li H, Liao LM, Zhang XM: **[Study on 1389 men who have sex with men regarding their HIV high-risk behaviors and associated factors in mainland China in 2004]**. *Zhonghua Liu Xing Bing Xue Za Zhi* 2007, **28**(1):32-36.

8. Choi KH, Ning Z, Gregorich SE, Pan QC: **The influence of social and sexual networks in the spread of HIV and syphilis among men who have sex with men in Shanghai, China**. *J Acquir Immune Defic Syndr* 2007, **45**(1):77-84.

9. Xu J, Zhang HB, Zheng YJ, Wang J, Zhu YB, Li ZR, Hu ZW: **[The prevalence of syphilis and HIV infection among young men who have sex with men in Hefei city]**. *Chinese Journal of Behavioral Medical Science* 2007, **16**(3):205-207.

10. Li X, Shi W, Li D, Ruan Y, Jia Y, Vermund SH, Zhang X, Wang C, Liu Y, Yu M *et al*: **Predictors of unprotected sex among men who have sex with men in Beijing, China**. *Southeast Asian J Trop Med Public Health* 2008, **39**(1):99-108.

11. Xu J, Wu ZJ, Zhang HB, Zhu JL, Wu HH, Zhu YB, Hu ZW, Ke WH, Zhang XP: **[Study On The Status Of HIV Infection And Syphilis Infection Among MSM In Hefei And Its Correlation With The Social And Sexual Partner Characteristics Of MSM]**. *Modern Preventive Medicine* 2007, **34**(16):3009-3011, 3014.

12. Zhang B, Li X, Chu Q, Wang n, Wang Z, Zhou S, Tong C, Zhang J, Guan W, Cui Z *et al*: **[A survey of HIV/AIDS related behaviors among 2250 MSM in nine major cities of China]**. *Chinese Journal of AIDS & STD* 2008, **14**(6):541-547.

13. Sun ZX, Lin SF, Wen MQ: **[Investigation on STD and AIDS prevalence for men who have sex with men]** *Modern Preventive Medicine* 2007, **34**(21):4130-4132.

14. Choi J, Xu QY, Huang YZ, Wang YN, Liu Y: **[A survey on AIDS knowledge and sexual behaviors among homosexual men in Nanchang city, Jiangxi Province]**. *China Medical Herald* 2007, **4**(17):158, 168.

15. Li N, Wang Z, Sun GQ, Sun DY: **[Analysis of HIV/AIDS sentinel surveillance among high risk population in Henan province in 2006]**. *Chinese Journal of AIDS & STD* 2007, **13**(5):427-429.

16. Fu LJ, Fang YR, Guo TY: **[Investigation of the sexual behaviors among the MSM in Shaoxing City of Zhejiang Province]**. *Disease Surveillance* 2007, **22**(12):818-819.

17. Ma J, Guo J: **[Internet survey of high risk sexual behaviors and sexually transmitted diseases among male homosexuals in Tianjin]**. *Modern Preventive Medicine* 2007, **34**(20):3928-3931.

18. Yang SJ, Mu HT, Li XQ, Li F, Zheng CJ, Zhang ZZ, Duo LK, Li RL, Shi L, Yang CX: **[Survey Of The Knowledge, Attitude, And Practice On AIDS Among MSM Population In Urumchi]**. *Modern Preventive Medicine* 2007, **34**(20):4624-4625.

19. Ouyang L, Ding XB, Zhou C, Lu RR: **[HIV risk sexual behavior among MSM with different sex orientation in Chongqin]**. *South China J Prev Med* 2008, **34**(2):16-19, 23.

20. Chen SH, Zhou J, Zhu JJ, Li KF: **[Analysis On Ethological Characters Of MSM In Nanning]**. *Modern Preventive Medicine* 2008, **35**(5):902-904.

21. Feng L, Ding X, Lu R, Liu J, Sy A, Ouyang L, Pan C, Yi H, Liu H, Xu J *et al*: **High HIV prevalence detected in 2006 and 2007 among men who have sex with men in China's largest municipality: an alarming epidemic in Chongqing, China**. *J Acquir Immune Defic Syndr* 2009, **52**(1):79-85.

22. Zhou J, Zhu JJ, Bin H, Zhang L, Yao M, Zhang W, Zhang W, Zhong JY, You QY, Gao L: **[A survey of HIV/STD,HBV and HCV infections and risk behaviors among MSM in two central districts of Guiyang city]**. *Chinese Journal of AIDS & STD* 2008, **14**(1):47-48, 51.

23. Liu H, Cai Y, Rhodes AG, Hong F: **Money boys, HIV risks, and the associations between norms and safer sex: a respondent-driven sampling study in Shenzhen, China**. *AIDS Behav* 2009, **13**(4):652-662.

24. Feng Y, Wu Z, Detels R, Qin G, Liu L, Wang X, Wang J, Zhang L: **HIV/STD prevalence among men who have sex with men in Chengdu, China and associated risk factors for HIV infection**. *J Acquir Immune Defic Syndr* 2010, **53 Suppl 1**:S74-80.

25. Zhao H, Wang G, Liu H: **[The Investigation Report of Male-to-male Contact During the Voluntary Consulation in Daolin District of Harebin]**. *Guide of China Medicine* 2009, **7**(10):181-183.

26. Feng LG, Ding XB, Xu j, Ou YL, Xu SM, Zheng JQ, Guo XJ, Yang MF, Liu XP: **[Study on HIV, Syphilis and HCV Prevalence and Its Assoicated Factors among Internet MSM Comparison to Non-Internet MSM in Chongqing]**. *Journal of Tropical Medicine* 2010, **10**(1):78-82.

27. Xu J, Han DL, Liu Z, Ma XY, Wang LL, Xu J, Pang L, Zhang HB, Wu ZY: **[The prevalence of HIV infection and risk factors among MSM in 4 cities, China]**. *Chin J Prev Med* 2010, **44**(11):975-980.

28. Liang L, Chen ZQ, Miao XF, Li BJ, Bai GY, Zhao HR: **[Investigation on AIDS knowledge and behaviors among men who have sex with men]**. *Hebei Medical Journal* 2009, **31**(19):2654-2655.

29. Wei C, Ruan S, Zhao J, Yang H, Zhu Y, Raymond HF: **Which Chinese men who have sex with men miss out on HIV testing?** *Sex Transm Infect* 2011, **87**(3):225-228.

30. Xu JJ, Reilly KH, Lu CM, Ma N, Zhang M, Chu ZX, Wang JJ, Yun K, Shang H: **A cross-sectional study of HIV and syphilis infections among male students who have sex with men (MSM) in northeast China: implications for implementing HIV screening and intervention programs**. *BMC Public Health* 2011, **11**(1):287.

31. Cai WD, Fen TJ, Tan JQ, Chen L, Shi XD, Chen PL, Jiang LZ, Tao XY: **[A Survery Of The Characteristics And STD/HIV Infection Of Homosexuality In Shenzhen]**. *Modern Preventive Medicine* 2005, **32**(4):328-330.

32. Zhang X, Wang C, Hengwei W, Li X, Li D, Ruan Y, Shao Y: **Risk factors of HIV infection and prevalence of co-infections among men who have sex with men in Beijing, China**. *AIDS* 2007, **21 Suppl 8**:S53-57.

33. Xiao Y, Ding X, Li C, Liu J, Sun J, Jia Y: **Prevalence and correlates of HIV and syphilis infections among men who have sex with men in Chongqing Municipality, China**. *Sex Transm Dis* 2009, **36**(10):647-656.

34. Lan Y, Gu Y, Wang B, Zhou D, Zhang J: **[Behavioral Features of Men Who Have Sex with Men]**. *Journal of Sichuan University (Medical Science Edition)* 2004, **35**(3):372-375.

35. Jiang SM, Li JZ, Li RY, Zhang HJ, Xiao HC: **[Investigation on unprotected sex and AIDS knowledge among men who have sex with men in Weihai city]**. *Prev Med Trib* 2006, **12**(6):763-764.

36. Zhang D, Bi P, Lv F, Zhang J, Hiller JE: **Changes in HIV prevalence and sexual behavior among men who have sex with men in a northern Chinese city: 2002-2006**. *Journal of Infectection* 2007, **55**(5):456-463.

37. Tao X, Zhou H, Cai Y, Cai W, Wei AY, Song D: **[114 examples male male contact AIDS high dangerous behavior investigates]** *Chinese Journal of Behavioral Medical Science* 2006, **15**(9):839-840.

38. He Q, Wu X, Han D, Liang X: **[HIV Infection and Risk Behavior of Men Having Sex With Men in Chengdu City From 2004 to 2007]**. *Journal of Occupational Health and Damage* 2008, **23**(4):222 - 224.

39. Tian XB, Ji YN: **[Analysis on condom use of men who have sex with men in small and medium city]**. *Chin J Public Health* 2006, **22**(11):1316-1317.

40. Xing JM, Zhang KL, Chen X, Zheng J: **[Investigation on HIV/AIDS knowledge and sexual behaviors among men who have sex with men in Hunan province]**. *Chin J Prev Med* 2007, **41**(6):511-513.

41. Chen SC, Luo Y, Cheng J, Ding JM, Dai YZ, Xu K, Chen KK, Chen WY, Shi SF: **[Analysis on results of MSM behavioral surveillance on HIV/AIDS]**. *Disease Surveillance* 2007, **22**(3):175-177.

42. Liao MZ, Liu XZ, Fu JH, Qian YS, Zhang XF: **[Analysis of data of behavioral surveillance in men who have sex with men(MSM) in Shandong Province]**. *Chin J AIDS STD* 2006, **12**(6):530-532.

43. Lai YH, Cai YM, Zheng XC, Liu H, Hong FC, Zhou H: **[STD/HIV Hish Risk Behavior Survey among Men Who Have Sex with Men in Shenzhen]**. *Southern China Journal of Dermato-Venereology* 2006, **13**(2):146-149.

44. Lu CG, Yuan F, Shi Z, Yang JZ, Li XY, Gao L, Li X, Hu SY: **[The study of HIV infection and KABP about AIDS among the MSM in Guiyang city]**. *Guizhou Medical Journal* 2006, **30**(3):202-204.

45. Lau JT, Wang M, Wong HN, Tsui HY, Jia M, Cheng F, Zhang Y, Su X, Wang N: **Prevalence of bisexual behaviors among men who have sex with men (MSM) in China and associations between condom use in MSM and heterosexual behaviors**. *Sex Transm Dis* 2008, **35**(4):406-413.

46. Chen SH, Zhou J, Zhu JJ: **[A analysis on MSM group behaviour character in Guangxi]**. *Journal of Applied Preventive Medicine* 2007, **13**(6):341-343.

47. Cai GF, Ma QQ, Pan XH, Fu LJ, Xu WX, Shan XR, Yang Q: **[HIV/AIDS Related Knowledge, Attitude, Practice and HIV/STD Infection among MSM in Two Cities of Zhejiang Province]**. *China Preventive Medicine* 2008, **9**(6):482-485.

48. Wang L, Tang HL, Zhang DP, Wu YH, Zhang J, Lv F: **[Behavioral features of men who have sex with men with different sexual orientations]**. *Chinese Journal of AIDS & STD* 2007, **13**(2).

49. Cai X: **[The Analysis of Risk Behaviors of MSM in Liaocheng and the Serological Detection for Anti-HIV, Anti-TP, and Anti-HCV Antibodies in 2006]**. *Preventive Medicine Tribune* 2007, **13**(10):888-890.

50. Chen S, Zhou J, Zhu JJ: **[Investigation of STI among some Men Who Have Sex with Men in Nanning City in 2006]**. *Prev Med Trib* 2007, **13**(9):772-774.

51. Wu J, Chen L, Fan HL, Ruan Y: **[A survey on the prevalence of HIV-1 and syphilis infection and characteristics of sexual behaviors in MSM (men who have sex with men) living in Shanghai]**. *J Diagn Concepts Pract* 2008, **7**(3):296-299.

52. Wang CH, Yang YH, Lu GJ, Yang XX, Ge LR, Zhao HR, Zhang YQ: **[Intervent male male sexual contact AIDS high dangerous behavior and evaluate effection of intervention]**. *Chinese Journal of Health Laboratory Technology* 2007, **17**(12):2291-2292.

53. Li Y, Chen JJ, Gu LP, Gao WL: **[Survey of HIV/SY Infection and KABP Related to AIDS among the MSM in Lanzhou]**. *Chinese Medical Science & Health* 2007, **8**:1-3.

54. Wang HL, Zhang M, Hu QH, Ding HB, Zhao B: **[Prevalence of HIV/STD and risk behavior among men who have sex with men in Shenyang]**. *Chin J Public Health* 2008, **24**(8):995-997.

55. Zeng G, Xiao Y, Xu P, Feng N, Jin CR, Lu F: **[Evaluation of effect of community-based HIV/AIDS interventions among men who have sex with men in eighteen cities, China]**. *Zhonghua Yu Fang Yi Xue Za Zhi* 2009, **43**(11):977-980.

56. Zhou J, Zhu JJ, Bin H, Zhang L, Yao M, Zhang W, Zhang W, Zhong JY, You QY, Gao L: **[A survey of HIV/STD,HBV and HCV infections and risk behaviors among MSM in two central districts of Guiyang city]**. *Chin J AIDS STD* 2008, **14**(1):47-48, 51.

57. Li N, Wang Z, Sun GQ, Sun DY: **[Analysis of HIV/AIDS sentinel surveillance among high risk population in Henan province in 2006]**. *Chin J AIDS STD* 2007, **13**(5):427-429.

58. Duan YW, Zhang HB, Wang XD, Wang Y, Jiang HY, Wang J: **[Survey on HIV related risk factors among MSM in Mianyang and Yibin]**. *Chin J Dis Control Prev* 2010, **14**(12):1189-1192.

59. China Global Fund AIDS Program Round 5: **[Chongqing Global Fund AIDS Program - Annual Assessment Report]**. In*.*; 2007.

60. Liu LY: **[Investigation on HIV/AIDS sexual behavior among men who have sex with men in 2006 in city of Mudanjiang]**. *Med Ani Prev* 2008, **24**(7):534-535.

61. Chen SH, Zhu JQ, Yang NH: **[Investigation on HIV and STI Infections among Men Who Have Sex with Men in Nanning City during 2006-2008]**. *Occipation and Health* 2010, **26**(1):56-58.

62. Xu ZH: **[Investigation on AIDS knowledge and behaviors among men who have sex with men in the city of Tieling]**. *Chin J Public Health* 2009, **25**(2).

63. Xu YF, Zhou J, Chen SH, Zhou FH, Mo JC, Zhu JJ: **[Investigation of HIV Knowledge and High-risk Behavior Among Men Who Have Sex With Men in Nanning]**. *Journal of Preventive Medicine Information* 2008, **24**(10):753-755.

64. Guo Y, Zhu XK, Xia JH, Dong XY: **[Study on HIV/syphilis infections among men who have sex with men and their behavioral feature]**. *Chin J AIDS STD* 2009, **15**(1):50-51, 71.

65. Zou H, Wu Z, Yu J, Li M, Ablimit M, Li F, Pang L, Juniper N: **Sexual risk behaviors and HIV infection among men who have sex with men who use the internet in Beijing and Urumqi, China**. *J Acquir Immune Defic Syndr* 2010, **53 Suppl 1**:S81-87.

66. Zeng H, Ding XB, Xu JW, Lu RR, Zhang L, Zhang ZJ, Wang Y: **[Status and demand on HIV/AIDS control among men who have sex with men in main districts of Chongqing]**. *Academic Journal of Second Military Medical University* 2011, **32**(5):494-499.

67. Cai YM, Liu H, Pan P, Hong FC, Feng TJ: **[Survey of HIV/AIDS related knowledge and high risk behavior among men who have sex with men in Shenzhen using respondent-driven sampling]**. *South China J Prev Med* 2009, **35**(1):4-7.

68. Guo LY: **[Survey of KAP towards AIDS in MSM]**. *China Tropical Medicine* 2008, **8**(4):634-636.

69. Weng YQ, Bai Y: **[Surveillance on the high risk behaviors among 239 men who have sex with men]**. *Journal of Applied Preventive Medicine* 2009, **15**(3):152-153.

70. Zhou SJ, Pan CB, Meng XR, Chen GQ, Zhang BC: **[The related research on sexual behavior and HIV infection in MSM of Chongqing]**. *Chin J Infect Control* 2008, **7**(6):381-384, 380.

71. Guo W, Song AJ, Meng HD, Pang L, Rou KM, Wu ZY: **[Survey on AIDS/STD risk behaviors and prevalence among men who have sex with men in Langfang, Hebei]**. *Chinese Journal of Epidemiology* 2008, **29**(6):545-547.

72. Sun M, Li D, Jin W, Jiang J, Guan L: **[Investigation on the Infection of HIV, HCV, Syphilis and HBV among MSM in Dalian City in 2008]**. *Preventive Medicine Tribune* 2009, **15**(11):1074 - 1075.

73. Ding XB, Feng LG, Xu J, Xu SM, Guo Xj, Zheng JQ, Yang MF, Liu XP: **[Study on the prevalence of HIV, Syphilis, HCV and HSV-II and its associated factors among 743 men who have sex with men in Chongqing]**. *Chinese Journal of Disease Control & Prevention* 2010, **14**(3):227-231.

74. Li LD, Zhao WB, Jiang YF: **[Study on HIV/AIDS infection and associated factors among men who have sex with men in Anshan, Liaoning province]**. *Chinese Medical Journal of Metallurgical Industry* 2010, **27**(6):684-685.

75. Miao ZF, Li J, Lei LM, Han X, Zhang XP: **[Survey of AIDS-related Knowledge and Behavior in 312 MSM]**. *Journal of Ningxia Medical University* 2009, **31**(6):761-762.

76. Chen SH, Zhu Jj, Li J: **[Investigation on knowledge of HIV/AIDS and related behaviors character among men who have sex with men in Nanning, Guangxi]**. *Chin J Dis Control Prev* 2010, **14**(2):130-133.

77. Li X, Xing AH, Chang WH, Cui M, Jia H, Ren Q, Lu Y, Wang BS, Zhang JJ: **[Analysis on AIDS surveillance on MSM in Xi'an,2003-2008]**. *Chin J Dis Control Prev* 2009, **13**(4):442-444.

78. Shi WD, Li G, Yang T, Zhou W, Liu PL, Li SL, Chen L, Wei J: **[Survey of High Risk Sexual Behaviors and HIV,Syphilis,HCV among 456 Male Homosexuals in Wuhan]**. *Medicine and Society* 2009, **22**(10):42-43.

79. Zhang M, Wang XD, Yang Y: **[Prevalence oh HIV, anti-HCV, syphilis infection and AIDS knowledge among men who have sex with men (MSM) in Urumqi]**. *Chin J Public Health* 2009, **25**(9):1075-1076.

80. Qu L, Yang JY, Zhang XG, Yang YR, Bao ZQ: **[Study on HIV and syphilis infections among men who have sex with men in selected cities of Inner Mongolia]**. *Chin J Epidemiol* 2009, **30**(10).

81. Wang XL, He JK, Su HH, Cao HZ, Zhang QJ, Zhao GL: **[HIV and syphilis infection in male homosexuality in Tangshan city]**. *Chinese Journal of Public Health* 2009, **25**(7):787-788.

82. Wang M, Deng YH, Dong HY, Duan Y: **[Behavior surveillance among men who have sex with men in Kunming in 2008]**. *Soft Science of Health* 2009, **23**(5):593-598.

83. Ma YG, Ya X: **[Investigation on Status of HIV Infection and High-risk Behaviors among Who Have Sex With Men in Some Cities of Tibet]**. *Chinese Journal of Social Medicine* 2010, **27**(4):254-255.

84. Wang ZJ, Sun L, Ma XJ: **[Survey on AIDS/STD risk behaviors and prevalence among men who have sex with men in Guangling,Yangzhou]**. *Jiangsu Journal of Preventive Medicine* 2010, **21**(2):4-7.

85. Gong CT, Zhang QH: **[Results of AIDS monitoring of 252 MSM in Quanzhou City]**. *China Tropical Medicine* 2010, **10**(12):1496-1497.

86. Liu PL, Yao ZZ, Shi WD, Ding J, Li SL, Chen L, Yang T, Wang X, Zhou W: **[Epidemiological study on the status of HIV/STD among MSM in Wuhan City]**. *Chin J Dis Control Prev* 2010, **14**(9):917-919.

87. Wang T, Lai XH, Li L, Chen CY, He BH: **[Survey on AIDS/STD Risk Behaviors and Prevalence Among Men Who Have Sex with Men in Zhongshan, Guangdong]**. *Practical Preventive Medicine* 2010, **17**(7):1261-1263.

88. Zhang JM: **[Investigation on the health status among 110 men who have sex with men in Jiangshui County, Yunnan Province]**. *Health World* 2010, **4**(11):37-38.
